# Protocol for a randomised controlled trial of standard wound management versus negative pressure wound therapy in the treatment of adult patients with an open fracture of the lower limb: UK Wound management of Open Lower Limb Fractures (UK WOLFF)

**DOI:** 10.1136/bmjopen-2015-009087

**Published:** 2015-09-22

**Authors:** Juul Achten, Nick R Parsons, Julie Bruce, Stavros Petrou, Elizabeth Tutton, Keith Willett, Sarah E Lamb, Matthew L Costa

**Affiliations:** 1 Warwick Medical School, The University of Warwick, Coventry, UK; 2 Nuffield Department of Orthopaedics, Rheumatology & Musculoskeletal Sciences, University of Oxford, Oxford, UK

**Keywords:** ACCIDENT & EMERGENCY MEDICINE, WOUND MANAGEMENT, ORTHOPAEDIC & TRAUMA SURGERY

## Abstract

**Introduction:**

Patients who sustain open lower limb fractures have reported infection risks as high as 27%. The type of dressing applied after initial debridement could potentially affect this risk. In this trial, standard dressings will be compared with a new emerging treatment, negative pressure wound therapy, for patients with open lower limb fractures.

**Methods and analysis:**

All adult patients presenting with an open lower limb fracture, with a Gustilo and Anderson (G&A) grade 2/3, will be considered for inclusion. 460 consented patients will provide 90% power to detect a difference of eight points in the Disability Rating Index (DRI) score at 12 months, at the 5% level. A randomisation sequence, stratified by trial centre and G&A grade, will be produced and administered by a secure web-based service. A qualitative substudy will assess patients’ experience of giving consent for the trial, and acceptability of trial procedures to patients and staff. Patients will have clinical follow-up in a fracture clinic up to a minimum of 12 months as per standard National Health Service (NHS) practice. Functional and quality of life outcome data will be collected using the DRI, SF12 and EQ-5D questionnaires at 3, 6, 9 and 12 months postoperatively. In addition, information will be requested with regards to resource use and any late complications or surgical interventions related to their injury. The main analysis will investigate differences in the DRI score at 1 year after injury, between the two treatment groups on an intention-to-treat basis. Tests will be two sided and considered to provide evidence for a significant difference if p values are less than 0.05.

**Ethics and dissemination:**

Ethical approval was given by NRES Committee West Midlands—Coventry & Warwickshire on 6/2/2012 (ref: 12/WM/0001). The results of the trial will be disseminated via peer-reviewed publications and presentations at relevant conferences.

**Trial registration number:**

ISRCTN33756652.

**Strengths and limitations of this study**

- This will be the first multicentre randomised trial to assess the clinical and cost-effectiveness of negative pressure wound therapy for open lower limb fractures.
- Methodological qualities of the trial include: large number of intervention sites, optimised protocol to reduce risk of bias, appropriate sample size calculation and planned intention-to-treat analysis.
- The challenge for this study will be the emergent nature of treatment for these patients and the temporary lack of capacity often experienced by this group of patients in the immediate period after the open fracture.

## Introduction

Fractures of the lower limb are extremely common injuries in the civilian and military populations. Fortunately, the majority of these injuries are ‘closed’, that is, the skin around the fracture is intact. In such cases, the risk of infection is low. However, if the fracture is ‘open’ such that the barrier provided by the skin is breached, then the broken bone is exposed to contamination from the environment.

In open fractures, the risk of infection is greatly increased.^1^ Wounds associated with open fractures of the lower limb are graded by severity, as part of routine clinical practice, using the classification of Gustilo and Anderson;^2^ grade 1 injuries are small wounds (a laceration less than 1 cm), grade 2 involve larger wounds (laceration greater than 1 cm) but without extensive soft-tissue damage, and grade 3 wounds have a laceration greater than 1 cm with extensive soft-tissue damage. In addition, Gustilo and Anderson described a special type of grade 3 injury that involved damage to a major blood vessel that required surgical repair. The greater the extent of the injury to the soft-tissues around the broken bone, the greater the risk of infection.^2^ In severe, high-energy fractures of the lower limb, infection rates of 27% are still reported, even in specialist trauma centres.^3^

If complications, such as surgical site infection occur, treatment frequently continues for years after the open fracture. There is a huge healthcare cost associated with such injuries (US study: $163 000 if the limb can be salvaged and $500 000 + if amputation is required), and this is a fraction of the subsequent personal and societal cost.^4^ In the UK civilian population, the risk of an open long-bone fracture is approximately 11.5/100 000/year,^5^ but this is much higher in the military population and the severity of the injuries frequently greater.^6^

The initial management of open fracture of the lower limb in the emergency department involves the removal of gross contamination, the application of a sealed dressing and the administration of antibiotics, as described in the joint British Orthopaedic Association/British Association of Plastic Reconstructive and Aesthetic Surgeons publication ‘standards for the management of open fractures of the lower limb’. (http://www.boa.ac.uk/site/show publications.aspx?id=59). Some patients may be transferred immediately to a hospital with specialist facilities (Major Trauma Centre). However, the key component of the management is the surgical ‘debridement’—removal of all contaminated tissue and washout of the open fracture in the operating theatre. Once the wound is clean, the fracture is usually immobilised with some form of internal or external fixation and a dressing is applied. This proposal concerns the type of dressing that is applied to the wound at the end of the operation.

Traditionally, a non-adhesive layer is applied to the exposed area. This is then covered with a sealed dressing or bandage to protect the open fracture from further contamination. The wound is covered in this way until a second look is done and further debridement is performed in the operating theatre, usually 48 h after the initial injury. This method has been used throughout the National Health Service (NHS) and in military practice for many years. However, any bleeding or ooze from the open fracture will collect under or on the dressings; this may be uncomfortable for the patient and may pose an infection risk.

Negative-pressure wound therapy (NPWT) is an alternative form of dressing which may be applied to open fractures. In this treatment, an ‘open-cell’, solid foam is laid onto the wound followed by a sealed dressing. A hole is made in the dressing overlying the foam and a sealed tube is used to connect the foam to a pump which creates a partial vacuum over the wound. This negative-pressure therapy removes blood and ooze from the area of the wound, may also remove any bacteria left in the wound and encourages the formation of ‘granulation’ (healing) tissue.^7^ Recent laboratory studies have also suggested that NPWT may stimulate the release of ‘cytokines’ that encourage new blood vessel formation.^8^ However, NPWT is considerably more expensive than traditional wound dressings, for the dressing and the associated machinery which generates the partial vacuum.

NPWT has shown encouraging results in clinical trials related to diabetic foot wounds^9^ and abdominal wounds,^10^ but there is only one randomised trial comparing standard wound dressing with NPWT for patients with open fractures of the lower limb.^11^ This trial demonstrated a reduction in the rate of wound infection in the group of patients treated with NPWT. However, the study had relatively small numbers (59 patients, 63 fractures), was single-centre, included only the most severe types of injury and was funded by a commercial company which produces a NPWT system. There are no similar trials registered on the international trials database.

Despite the limited supporting evidence, the current British Orthopaedic/British Association of Plastic Surgery guidelines (http://www.boa.ac.uk/site/showpublications.aspx?id=59) for the management of open fractures of the lower limb already include reference to the use of NPWT. A recent consensus paper, published by the International Expert Panel on NPWT (NPWT-EP)^12^ also recommended that NPWT “should be considered when primary closure is not possible” in the management of wounds associated with open fractures, but acknowledged that the evidence base to support this statement was very limited.

We believe that there is a pressing need to evaluate this relatively expensive technology. We, therefore, propose a multicentre randomised clinical trial comparing negative pressure wound therapy with standard dressings for patients with wounds associated with open fractures of the lower limb.

## Methods

The trial will be conducted in accordance with the Medical Research Council's Good Clinical Practice (MRC GCP) principles and guidelines, the Declaration of Helsinki, Warwick Clinical Trials Unit (CTU) SOPs, relevant UK legislation and the Protocol. GCP-trained personnel will conduct the trial. The trial will be reported in line with the CONSORT statement.

### Trial summary

The proposed project is a two-phased study. Phase 1 (feasibility phase) will assess the feasibility of running a large-scale multicentre randomised controlled trial in this complicated area of trauma research. Phase 2 (main phase) will consist of conducting the proposed randomised controlled trial in a minimum of 20 trauma centres across the UK.

#### Feasibility summary

The feasibility phase will take place in five centres over a period of 6 months. The main trial will run, as described below, with the addition of a qualitative substudy assessing patients’ experience of giving consent for the trial and the acceptability of the trial procedures to patients and staff. Screening logs will be kept at each site to determine the number of patients assessed for eligibility and reasons for any exclusion. In addition, the number of eligible and recruited patients, and the number of patients who withdraw will be recorded.

#### Main randomized controlled trial summary

All adult patients presenting at the trial centres within 72 h of sustaining an open fracture of the lower limb are potentially eligible to take part in the trial. Inclusion within the trial depends on the severity of the wound associated with the fracture. Gustilo and Anderson grade 2 and 3 injuries will be included.

A randomisation sequence, stratified by trial centre and Gustilo and Anderson grade, will be produced and administered by a secure web-based service. The random allocation will be to either standard wound management or negative pressure wound therapy.

The patients will have clinical follow-up in the local fracture clinic up to a minimum of 12 months as per standard NHS practice after this injury. Functional and quality of life outcome data will be collected using the DRI, SF12 and EQ-5D questionnaires at 3, 6, 9 and 12 months postoperatively. These questionnaires will be administered centrally by a data administrator via post or telephone, or these will be collected during routine clinic appointments by the local research associate. In addition, at the same time points, information will be requested with regards to resource use and any late complications or surgical interventions related to their injury with a specific note of continuing treatment for the deep infection.

### Null hypothesis

There is no difference in the Disability Rating Index score (DRI) 1-year postinjury between adult patients with an open fracture to the lower limb treated with standard wound dressings versus negative pressure wound therapy before definitive wound closure.

### Objectives

This pragmatic randomised controlled trial will compare standard dressings with negative pressure wound therapy in the treatment of wounds associated with open fractures of the lower limb.

The specific objectives for the feasibility phase of this study are:

- *FEAS 1:* A qualitative assessment of patients’ experience of sustaining a fracture of the lower limb, being enrolled in the study giving or declining consent for the trial and the acceptability of the trial procedures to patients and staff.
- *FEAS 2:* To determine the number of *eligible, recruited and withdrawn patients* in the 5 feasibility trauma centres over the course of 6 months. In addition, to determine if any of the trial patients lack capacity to give consent 6 weeks postinjury.

At the end of the feasibility phase, the Trial Management Group will provide a report to the Trial Steering Committee (TSC). The report will show the actual rate of recruitment at the five centres involved in the feasibility phase compared with the target rate of recruitment (one patient per month per centre), in the context of the results of the qualitative study. If the patients are willing to give their consent and the rate of recruitment achieves the target rate by the end of the feasibility phase, we would anticipate proceeding to the main trial.

The primary objective for the full randomized controlled trial (RCT) is:

- *MAIN 1*: To quantify and draw inferences on observed differences in the DRI at 12 months after the open fracture.

The secondary objectives are:

- *MAIN 2*: To quantify and draw inferences on patient-reported differences in ‘deep infection’ of the limb, in the 12 months after the open fracture. Photographs will be used to assess wound healing. Any infection that requires continuing medical intervention or has already led to amputation at or after the six-week review will be considered a ‘deep’ infection.
- *MAIN 3*: To quantify and draw inferences on observed differences in general quality of life (SF-12 and EQ-5D) in the 12 months after the open fracture.
- *MAIN 4*: To determine the number and nature of further surgical interventions related to the injury, during the first 12 months after the open fracture.
- *MAIN 5*: To investigate, using appropriate statistical and economic analysis methods, the resource use and thereby, the cost-effectiveness of negative pressure wound therapy versus standard dressing for wounds associated with open fractures of the lower limb.

### Outcome measures

The primary outcome measure for this study is the *DRI* a self-administered, 12-item Visual Analogue Scale questionnaire assessing the patients’ own rating of their disability.^13^ This measure was chosen as it addresses ‘gross body movements’ rather than specific joints or body segments. Therefore, it will facilitate the assessment of patients with different fractures of the lower limb.

The secondary outcome measures in this trial are:

***Deep Infection****;* We will use the *Centres for Disease Control and Prevention* definition of a ‘deep surgical site infection’: that is, a wound infection involving the tissues deep to the skin that occurs in the first year following the injury.^14^ Any infection that requires continuing medical intervention or has already led to amputation at or after the routine 6-week outpatient appointment will be considered a deep infection.

We will use photographs of the wound at the 6-week clinical follow-up in order to provide an objective assessment of wound healing and infection. X-rays taken at 6 weeks and 12 months postinjury will be assessed for further indicators of infection—periosteal reaction/lysis at 6 weeks and chronic osteomyelitis at 12 months postinjury. The photographs and X-rays will be reviewed by two independent experienced assessors who are blind to the treatment allocation. In addition, patients will be asked to self-report on any further signs of infection and on any medical/surgical intervention related to infection associated with their open fracture at each of the follow-up time points.

***EuroQol EQ-5D*:** The EuroQol EQ-5D is a validated measure of health-related quality of life, consisting of a five dimension health status classification system and a separate visual analogue scale.^15^ Responses to the health status classification system will be converted into multiattribute utility (MAU) scores using a published utility algorithm.^16^ These MAU scores will be combined with survival data to generate quality-adjusted life year (QALY) profiles for the purposes of the economic evaluation.

***SF-12*:** The Short-Form 12 is a validated and widely used health-related quality of life measure (21). Each permutation of response to the SF-12 will be converted into a MAU score using a published utility algorithm.^17^ These data will be combined with survival data to generate QALY profiles for the purposes of the economic evaluation.

***Complications***; all complications and surgical interventions related to the open fracture will be recorded.

***Resource use*** will be monitored for the economic analysis. Unit cost data will be obtained from national databases such as the BNF and PSSRU Costs of Health and Social Care (20). Where these are not available, the unit cost will be estimated in consultation with the UHCW finance department. The cost-consequences following discharge, including NHS costs and patients’ out-of-pocket expenses, will be recorded via a short questionnaire which will be administered at 3, 6, 9 and 12 months postsurgery. Patient self-reported information on service use has been shown to be accurate in terms of the intensity of use of different services.^18^

We will use techniques common in long-term cohort studies to ensure minimum loss to follow-up such as collection of multiple contact addresses and telephone numbers, mobile telephone numbers and email addresses. Considerable efforts will be made by the trial team to keep in touch with patients throughout the trial by means of newsletters etc.

### Sample size

The minimum clinically important difference (MCID) for the primary outcome measure (DRI) is assumed to be eight points.^13^ The DRI is a 12-item, patient-reported, functional outcome questionnaire that is transformed to a 100 point scale, where 0 represents normal function and 100 complete disability. At an individual patient level, a difference of eight points represents the ability to climb stairs or run with ‘some difficulty’ versus with ‘great difficulty’. At a population level, eight points represents the difference between a ‘healthy patient’ and a ‘patient with a minor disability’.

In table 1, the bold figure of 412 patients represents a conservative scenario, based on a SD of 25 and 90% power to detect the selected MCID. However, a sample size of 308 patients would still provide 80% power. Allowing a margin of 10% loss during follow-up, including the small number of patients who die in the first year following their injury, this gives a figure of *460* patients in total. Therefore, 230 patients who consent to each group will provide 90% power to detect a difference of eight points in DRI at 12 months at the 5% level.

**Table 1:** Sample size for varying power and SD

SD	Power	
	80%	90%
15	112	150
20	198	264
25	308	**412**

The sample size used in the trial is shown in bold.

### Methodology

#### Eligibility

Patients will be eligible for this study if:

- They are aged 16 years or older
- Present to the trial hospital within 72 h of injury
- Have an open fracture of the lower limb—graded as Gustilo and Anderson 2 or 3.

Patients will be included if they are transferred from another hospital to a trial centre within 72 h of their injury. (A very small number of patients may present after 72 h, but there is a possibility that any wound would already be infected with later presentations).

Patients will be excluded from participation in this study if:

- There are contra-indications to anaesthesia such that the patient is unable to have surgery
- There is evidence that the patient would be unable to adhere to trial procedures or complete questionnaires, such as permanent cognitive impairment. It is expected that for a very small proportion of patients this exclusion criterion will only be determined after randomisation has taken place. These patients will then be excluded from the study and no patient identifiable data will be retained.

Patients who sustain other injuries which may affect the primary outcome measure will have their injuries documented, but will be included in the analysis.

#### Recruitment and consenting

The feasibility phase of the study will specifically inform and test the recruitment rate for the main trial as well as assess the acceptability of the process of consent. Recruitment will take place in five trial centres over a period of 6 months for the feasibility phase. The expected rate of recruitment is based on recent audit data from two of the centres (Oxford and Coventry). In these centres, an average of four eligible patients are admitted with an open fracture to the lower limb every month. All centres involved in the trial will be Major Trauma Centres or Trauma Units with similar catchment areas as the five initial sites. During the main phase of the trial, trial site recruitment of the remaining sites will occur over a period of 8 months. Recruitment in these sites will take place over a period of 27 months to reach the target of a minimum of 460 patients.

Patients will be screened from the emergency department at the trial centres. All patients with an open fracture of the lower limb will be screened for eligibility by a research associate.

The nature of these injuries means that a majority of patients will be operated on immediately or be on the next available trauma operating list, depending on access to an appropriate operating theatre. A small number of patients are transferred between hospitals or wait for an operation, and could potentially give consent prior to randomisation. These are the patients who may wait up to 72 h before surgery.

Some patients may be unconscious, all will be distracted by the injury to their leg and its subsequent treatment, and all will have had large doses of opiates for pain relief, affecting their ability to process information. The majority of patients will, therefore, lack capacity to make a decision about participation in a research project at this stage. In this emergency situation, the focus will be on informing the patient and any next of kin about immediate clinical care. There will be limited time for the patient, if they had capacity, or their next of kin to review trial documentation and make an informed decision about whether they would wish to participate.

Conducting research in this ‘emergency setting’ is regulated by the *Mental Capacity Act (MCA) 2005*. As patients are likely to lack capacity, as described above, and because of the urgent nature of the treatment limiting access to and appropriate discussion with personal consultees, we propose to act in accordance with section 32, subsection 9b of the MCA for following a process approved by the relevant research ethics committee. The patients who have surgery on the next available trauma operating theatre enter the study under presumed consent. We will not obtain consent prior to surgery, but will inform the patient and seek patient consent for continuation in the trial at the first appropriate time point in the postoperative period.

For those patients who are able to give consent before their operation, namely those who have been transferred or are waiting up to 72hrs for their operation, will be approached by the research team for consent into the study.

A small number of the patients who have had their surgery delayed may still not have capacity to give consent, for example, those who are unconscious. If the clinical team in charge of that patient’s care do not think that the patient is able to provide clinical consent for their operation, then the research team will approach a consultee for agreement to randomisation. The patient themselves will be approached for consent as soon as the clinical team deem that they have regained capacity following their operation.

The treating surgeon will determine the final grade of the open fracture at the end of the debridement of the wound as per routine practice in the operating theatre, and then patients will automatically be enrolled into the study via the online randomisation system.

For those patients who did not give consent prior to surgery, at the first appropriate time when the patient has regained capacity, the research associate will provide the patients with all of the study information. The patients will be given the opportunity to ask questions, and discuss the study with their family and friends. They will then be asked to provide written consent for continuation in the study.

Throughout the whole study, screening logs will be kept at each site to determine the number of patients assessed for eligibility and reasons for any exclusion. Patients who decline to continue to take part during the feasibility phase will be given the opportunity to discuss/inform the research team of their reasoning behind their decision not to take part.

Any new information that arises during the trial that may affect participants’ willingness to take part will be reviewed by the TSC; if necessary, this will be communicated to all participants. A revised consent form will be completed, if necessary.

#### Qualitative substudy

Within the feasibility study, a qualitative substudy will assess patient experience of having an open fracture of the lower limb, being enrolled in the study, giving or declining consent for the trial, and the acceptability of the trial procedures.

The sample will include patients at two UK sites (Coventry and Oxford). This will include standard wound care and negative pressure wound therapy. Semistructured interviews will be undertaken with up to 20 consecutive patients who provide informed consent for the interview during their hospital stay. Participants will be given information about the interview study and provide written consent. The interviews will be conversational in style^19^ and focus on three areas (1) the experience of open fracture of the lower limb, (2) the impact and acceptability of the trial procedures, and (3) the process of consent to a trial. Those who preferred not to take part in the trial will be asked to tell us about (1) the experience of open fracture of the lower limb, and (2) their thoughts and feelings about the trial. The key interview questions will be what is it like: to experience an open fracture; have an open wound and dressing/negative pressure wound therapy; to be part of a trial/prefer not to take part in a trial? These will be followed by prompts such as: tell me more about that; how did that affect you; how did you feel about that; what were you thinking at that point. The interviews will take place in hospital when the patients are well enough and feel able to take part in the interview. Where possible, these interviews would take place in a private area on the ward, but at the bedside is more likely due to the nature of the injury. Attention will be paid to privacy and dignity of the patient, and the interview will be stopped and reconvened if the patient is uncomfortable or feels that their privacy is being compromised.

Interviews will be performed with both patients who agree to continue in the trial and those who decline to be further involved. The research team is aware that ethically patients do not have to provide a reason for their choice and should not be coerced in anyway. However, in light of the limited knowledge in this area, the value of understanding what trauma patients’ think and feel about research in this context would be substantial and would help to inform the recruitment process in the main trial. The researcher would take an exploratory, non-judgemental stance while allowing the patient to tell their story. As interviews will take place with participant refusers after they have withdrawn from the trial, the interview cannot be construed as coercive in relation to the trial.

Two focus groups, 1 on each site with up to 12 staff, will be undertaken with staff involved in the management of the trial or the management of patients in the trial. This will include surgeons, emergency department staff, theatre staff, ward staff and research staff. The participants will consider the factors that facilitate and inhibit the daily process of running the trial. This will include optimal timing and method to approach the patient with the participant information. Focus groups are a good way to access a range of views on a topic and provide opportunities for debate and challenge within the group.^20^ Managing the dynamics of a group is important to ensure all participants have a chance to share their views and strong views are contained. Attention will be paid to this through the use of basic ground rules and good facilitation of the group. The focus group discussions will take place in a quiet room away from interruptions. The interviews and focus groups’ meetings will be digitally recorded and transcribed verbatim. Analysis will be line-by-line, identifying codes, building categories and themes by drawing on the work of Miles and Huberman.^21^ NVivo9, a software package for qualitative data, will be used to help with data management. The intention of the patient interviews is to understand how patients make sense of their treatment and to specifically address any issues related to their consent to participate. The focus groups will develop a greater understanding of the factors that facilitate and inhibit the process of the trial. The qualitative data will be used to provide indepth understanding of the process to augment the quantitative data.

#### Trial ID

When a patient enters the trial, sufficient non-identifiable details will be logged on a secure, encrypted, web-based system, provided by York CTU. Basic information, including the patient's initials, date of birth, gender and eligibility checks will be entered. The patient will then receive a trial ID that will be used on all trial documentation.

#### Randomisation

The allocation sequence will be generated by an independent randomisation centre—York CTU. Randomisation will be on a 1:1 basis, stratified by trial centre and Gustilo and Anderson grade—2, 3, or 3 with vascular injury requiring surgical repair. Eligibility for the trial is based on a wound of grade 2 or above, which will be established definitively at the end of the initial surgical debridement in the operating theatre as per routine clinical practice. Therefore, participants will be assigned to their treatment allocation at the end of the initial surgery, but before the wound dressing is applied. All modern operating theatres include a computer with web-access; so a secure, 24 h, web-based randomisation system will be used to generate the treatment allocation intraoperatively.

#### Post randomisation withdrawals/exclusions

Participants will be excluded in the postrandomisation phase if it is established that they would be unable to adhere to trial procedures or complete questionnaires.

Participants may decline to continue to take part in the trial at any time without prejudice. A decision to decline consent or withdraw will not affect the standard of care the patient receives.

Participants have three options for withdrawal:

1. Participants may withdraw from completing any further questionnaires, but allow the trial team to still view and record anonymously any relevant hospital data that is recorded as part of normal standard of care, that is, X-rays and further surgery information.
2. Participants can withdraw wholly from the study, but data obtained up to the point of withdrawal will be included in the final analysis of the study; thereafter, no further data will be collected for that participant.
3. Participants can withdraw wholly from the study and data collected up to the point of withdrawal will not be included in the final analysis.

Once withdrawn from the study, the patient will be advised to discuss their further care plan with their surgeon.

#### Blinding

As the wound dressings are clearly visible, the patients cannot be blinded to their treatment. In addition, the treating surgeons will also not be blind to the treatment, but will take no part in the postoperative assessment of the patients. The functional outcome data will be collected and entered onto the trial central database via postal questionnaire by a research assistant/data clerk in the trial central office.

In addition, we will use photographs of the wound at the 6-week clinical follow-up to provide an objective assessment of wound healing and infection. The photographs will be reviewed independently by two experienced assessors who are blind to the treatment allocation.

### Trial treatments

Patients with an open fracture of the lower limb usually have surgery on the next available trauma operating list. Some patients may be transferred to a Major Trauma Centre for definitive care—within the first 48 h of injury—but will still have their initial surgery as soon as possible. All patients will receive a general or regional anaesthetic. The wound associated with the fracture is ‘debrided’ (surgical decontamination and cleansed) in the operating theatre and the fracture is treated with either internal or external fixation. At the end of the initial operation, a dressing is applied to the wound. This trial will compare two types of wound dressings: standard dressing versus negative pressure wound therapy.

#### Treatment options

Standard dressing: The standard dressing for open fractures comprises a non-adhesive layer applied directly to the wound which is covered by a sealed dressing or bandage. The standard dressing does not use ‘negative pressure’. The exact details of the materials used will be left to the discretion of the treating surgeon as per their routine practice, but the details of each dressing applied in the trial will be recorded.

NPWT: The NPWT dressing uses an ‘open-cell’, solid foam which is laid onto the wound followed by an adherent, sealed dressing. A hole is cut in the layer over the foam and a sealed tube is used to connect the foam to a pump which creates a partial vacuum over the wound. The basic features of the NPWT are universal, but the exact details of the dressing will be left to the discretion of the treating surgeon. Again, the details of the dressings used will be recorded in the trial documentation.

Both groups of patients will then follow the normal postoperative management of patients with an open fracture of the lower limb. This will usually involve a ‘second-look’ operation after 48 h, where a further debridement is performed and the wound closed (with sutures or a soft-tissue graft, as necessary). Depending on the specific injury and according to the treating surgeons’ normal practice, the wound may be redressed again pending further surgery. Any further wound dressing will follow the allocated treatment until definitive closure/cover of the wound is achieved.

#### Rehabilitation

The rehabilitation will be recorded but left entirely to the discretion of the treating surgeon, as the type of injury will vary between patients.

#### Follow-up

Baseline, standardised radiographs will be copied onto CD from the hospital PACs (archiving) system. Copies of the baseline clinical report forms and CD will be delivered to the trial coordinating centre.

The research associate will make a record of any early complications at the routine 6-week follow-up appointment and take a photograph of the wound. This data will be returned to the trial coordinating centre, together with a copy of the routine 6-week follow-up radiograph. The number and timing of any subsequent follow-up appointments will be at the discretion of the treating surgeon. All patients will be reviewed at 12 months as per routine practice after this type of injury. Details of any late complications and copies of the 12-month radiographs will be sent to the trial coordinating centre.

The functional outcome data will be collected using questionnaires at 3, 6, 9 and 12 months postoperatively (see table 2). In addition to the DRI, the patients will be asked to fill out the EuroQol and SF-12 questionnaires, and a complications/further surgical interventions and health economics questionnaire. The 3, 6 and 9 months postoperative questionnaires will be sent to the patients by post, a process done centrally by a data clerk at the Warwick CTU. All of the outcome questionnaires can be completed over the phone if postal copies are not returned. Patients will be asked to complete their 12 months postoperative questionnaire during their routine follow-up appointment 1-year postoperation.

**Table 2:** Follow-up measures

Time point	Data collection
Baseline	DRI and SF-12 preinjury, EQ-5D preinjury and contemporary, routine radiographs of leg wound(s)
6 weeks	Complication records, radiographs, operative record, photograph of leg wound(s)
3 months	DRI, EQ-5D, SF-12 record of complications/rehabilitation or other interventions, and economics questionnaire
6 months	DRI, EQ-5D, SF-12 record of complications/rehabilitation or other interventions, and economics questionnaire
9 months	DRI, EQ-5D, SF-12 record of complications/rehabilitation or other interventions, and economics questionnaire
12 months	DRI, EQ-5D, SF-12 record of complications/rehabilitation or other interventions, and economics questionnaire, radiographs

DRI, Disability Rating Index.

### Adverse event management

Adverse events (AE) are defined as *any untoward medical occurrence in a clinical trial subject and which do not necessarily have a causal relationship with the treatment*. All AEs will be listed on the appropriate Case Report Form for routine return to the ‘WOLLF’ central office.

Serious AEs are defined as any *untoward and unexpected medical occurrence that:*

1. Results in death
2. Is life-threatening
3. Requires hospitalisation or prolongation of existing inpatients´ hospitalisation
4. Results in persistent or significant disability or incapacity
5. Is a congenital anomaly or birth defect
6. Any other important medical condition which, although not included in the above, may require medical or surgical intervention to prevent one of the outcomes listed.

All serious AEs (SAE) will be entered onto the SAE reporting form and faxed to the dedicated fax system at WMSCTU within 24 h of the investigator becoming aware of them. Once received, causality and expectedness will be confirmed by the Chief Investigator. SAEs that are deemed to be unexpected and related to the trial will be notified to the Research Ethics Committee (REC) within 15 days. All such events will be reported to the TSC and Data Monitoring Committee (DMC) at their next meetings.

SAEs that may be expected as part of the surgical interventions and which do not need to be reported to the main REC are: complications of anaesthesia or surgery (wound infection, bleeding or damage to adjacent structures such as nerves, tendons and blood vessels, delayed unions/non-unions, delayed wound healing, further surgery to remove/replace metal-work and thromboembolic events). All participants experiencing SAEs will be followed-up as per protocol until the end of the trial.

#### Risks and benefits

The risks associated with this study are predominantly the risks associated with the injury and the surgery: infection, bleeding and damage to the adjacent structures such as nerves, blood vessels and tendons. Participants in both groups will undergo surgery and will potentially be at risk from any/all of these complications. Allocation of the trial intervention will take place at the end of the initial surgery so that there is no difference between the groups in terms of surgical risk.

Both standard wound dressings and NPWT have been used widely in the civilian and military settings, and there are no specific risks associated with the use of either type of wound management—other than a potential reduction in the rate of wound complications, which is the focus of this trial.

### End of trial

The end of the trial will be defined as the collection of 1-year outcome data from the last participant.

### Oversight

We will institute a rigorous programme of quality control. Quality assurance checks will be undertaken by Warwick CTU to ensure integrity of randomisation, study entry procedures and data collection. The Warwick CTU has a quality assurance manager who will monitor this trial by conducting regular (yearly or more, if deemed necessary) inspections of the Trial Master File. Furthermore, the processes of consent taking, randomisation, registration, provision of information and provision of treatment will be monitored. A TSC and a DMC will be set up. Written reports will be produced for the TSC, informing them if any corrective action is required.

## Statistical analysis

### Feasibility study

At the end of the feasibility phase, the overall mean recruitment at the five selected centres for this phase of the study will be estimated (with a 95% CI) and compared to the target rate of one patient per month per centre. The estimated recruitment rate and the overall rate of withdrawn patients in the feasibility phase will inform the design and the decision to proceed to the main RCT.

### Main RCT

Standard statistical summaries (eg, medians and ranges or means and variances dependent on the distribution of the outcome) and graphical plots showing correlations will be presented for the primary outcome measure and all secondary outcome measures. Baseline data will be summarised to check comparability between treatment arms, and to highlight any characteristic differences between those individuals in the study, those ineligible and those eligible, but withholding consent.

The main analysis will investigate differences in the primary outcome measure, the DRI score at 1 year after injury, between the two treatment groups (standard wound dressings and negative pressure wound therapy) on an intention-to-treat basis. In addition, early functional status will also be assessed and reported at 3, 6 and 9 months. Differences between groups will be assessed, based on a normal approximation for the DRI score at 12 months postinjury and at interim occasions. Tests will be two sided and considered to provide evidence for a significant difference if p values are less than 0.05 (5% significance level).

The stratified randomisation procedure should ensure a balance in Gustilo and Anderson grade and the recruiting centre's between-test treatments. Although generally we have no reason to expect that clustering effects will be important for this study, in reality, the data will be hierarchical in nature, with patients naturally clustered into groups by the recruiting centre. Therefore, we will account for this by generalising the conventional linear (fixed effects) regression approach to a mixed effects modelling approach, where patients are naturally grouped by recruiting centres (random effects). This model will formally incorporate terms that allow for possible heterogeneity in responses for patients due to the recruiting centre, in addition to the fixed effects of the treatment groups, Gustilo and Anderson grade, and other patient characteristics that may prove to be important moderators of the treatment effect such as age and gender.

It seems likely that some data may not be available due to voluntary withdrawal of patients, lack of completion of individual data items or general loss to follow-up. Where possible, the reasons for data ‘missingness’ will be ascertained and reported. Although missing data is not expected to be a problem for this study, the nature and pattern of the missingness will be carefully considered—including, in particular, whether data can be treated as missing completely at random. If judged appropriate, missing data will be imputed, using the multiple imputation facilities available in R (http://www.r-project.org/). The resulting imputed datasets will be analysed and reported, together with appropriate sensitivity analyses. Any imputation methods used for scores and other derived variables will be carefully considered and justified. Reasons for ineligibility, non-compliance, withdrawal or other protocol violations will be stated and any pattern observed will be summarised. More formal analysis, for example, using logistic regression with ‘protocol violation’ as a response, may also be appropriate and aid interpretation. About 1–2% of patients are expected to die during follow-up; so this is unlikely to be a serious cause of bias. However, we will conduct a secondary analysis taking account of the competing risk of death, using methods described by Varadhan *et al*.^22^

The main analyses will be conducted using specialist mixed effects modelling functions available in the software package R (http://www.r-project.org/) where DRI data will be assumed to be normally distributed, possibly after appropriate variance stabilising transformation. The primary focus will be the comparison of the two treatment groups of patients, and this will be reflected in the analysis which will be reported together with appropriate diagnostic plots that check the underlying model assumptions. Results will be presented as mean differences between the trial groups, with 95% CIs.

Secondary analyses will be undertaken using the above strategy for approximately normally distributed outcome measures SF-12 and EQ5D. For dichotomous outcome variables, such as indicators of deep infection and other complications related to the trial interventions, mixed effects logistic regression analysis will be undertaken with results presented as ORs (and 95% CIs) between the trial groups. Also, temporal patterns of any complications will be presented graphically and if appropriate, a time-to-event analysis (Kaplan-Meier survival analysis) will be used to assess the overall risk and risk within individual classes of complications.

A detailed statistical analysis plan (SAP) will be agreed on with the DMC. Any subsequent amendments to this initial SAP will be clearly stated and justified. Interim analyses will be performed only where directed by the DMC. The routine statistical analysis will mainly be carried out using R (http://www.r-project.org/) and S-PLUS (http://www.insightful.com/*).* Results from this trial will also be compared with results from other trials.

### Economic evaluation

An economic evaluation will be integrated into the trial design. The economic evaluation will be conducted from the recommended NHS and personal social services perspective.^23^ Data will be collected on the health and social service resources used in the treatment of each trial participant during the period between randomisation and 12 months postrandomisation. Trial data collection forms will record the duration of each form of hospital care, surgical procedures, adjunctive interventions, medication profiles and tests and procedures. Observational research may be required to detail additional staff and material inputs associated with clinical complications. At 3, 6, 9 and 12 months postrandomisation, trial participants will be asked to complete economic questionnaires profiling hospital (inpatient and outpatient) and community health and social care resource use, and for the purposes of sensitivity analysis, out-of-pocket expenditures and costs associated with lost productivity. Current UK unit costs will be applied to each resource item to value total resource use in each arm of the trial. *Per diem* costs for hospital care, delineated by level or intensity of care, will be calculated by the health economics researcher using data from detailed questionnaires completed by the local finance departments, giving cost data and apportioning these to different categories of patient using a ‘top-down’ methodology. The unit costs of clinical events that are unique to this trial will be derived from the hospital accounts of the trial participating centres, although primary research that uses established accounting methods may also be required. The unit costs of community health and social services will largely be derived from national sources, although some calculations from first principles using established accounting methods may also be required.^24^ Trial participants will be asked to complete the EuroQol EQ-5D^15^ and SF-12^25^ ^26^ measures at 3, 6, 9 and 12 months postrandomisation. Responses to the EQ-5D and SF-12 will be converted into MAU scores using established algorithms.^16^ ^17^

An incremental cost-effectiveness analysis, expressed in terms of incremental cost per QALY gained, will be performed. Results will be presented using incremental cost-effectiveness ratios (ICERs) and cost-effectiveness acceptability curves (CEACs) generated via non-parametric bootstrapping. This accommodates sampling (or stochastic) uncertainty and varying levels of willingness to pay for an additional QALY. Owing to the known limitations of within-trial economic evaluations,^26^ we will also construct a decision-analytical model to model beyond the parameters of the proposed trial the cost-effectiveness of negative pressure wound therapy in this clinical population. The model will be informed partly by data collected as part of the proposed trial, and also by data collected from other primary and secondary sources, including data sets held by the research team. Long-term costs and health consequences will be discounted to present values using discount rates recommended for health technology appraisal in the UK.^23^ A series of probabilistic sensitivity analyses will be undertaken to explore the implications of parameter uncertainty on the ICERs. Probabilistic sensitivity analyses will also explore the effects of extending the study perspective, target population, time horizon and decision context on the ICERs. In addition, CEACs will be constructed using the net benefits approach.

## Discussion

This pragmatic, multicentre trial is due to deliver results in Spring 2017. Results will be disseminated through peer-reviewed publications, including a National Institute for Health Research Health Technology Assessment monograph. Participants of the trial will receive a lay summary of the trial results.
